# Cleavage speed and implantation potential of early-cleavage embryos in IVF or ICSI cycles

**DOI:** 10.1007/s10815-012-9777-z

**Published:** 2012-07-25

**Authors:** Meng-Ju Lee, Robert Kuo-Kuang Lee, Ming-Huei Lin, Yuh-Ming Hwu

**Affiliations:** 1 Division of Reproductive Endocrinology and Infertility, Department of Obstetrics and Gynecology, Mackay Memorial Hospital, 92, Sec. 2, Chung San North Road, Taipei, 10449 Taiwan; 2 Division of Reproductive Endocrinology and Infertility, Department of Obstetrics and Gynecology, Mackay Memorial Hospital, Hsinchu, No. 690, Sec. 2, Guangfu Rd, East Dist, Hsinchu, 30071 Taiwan

**Keywords:** Early cleavage, Embryonic cleavage speed, Embryonic quality, Implantation rate, Live birth rate

## Abstract

We examined whether there is a correlation among early embryo cleavage, speed of cleavage, and implantation potential for in-vitro fertilization (IVF) treatment and intracytoplasmic sperm injection (ICSI). This retrospective study examined 112 cycles of IVF and 82 cycles of ICSI in patients less than 40 years of age. Early cleavage was defined as embryonic mitosis occurring 25–27 h after insemination. These day-3 embryos were then grouped according to cleavage speed (rapid, normal, and slow) and morphological quality (good or poor). A larger proportion of early-cleavage embryos developed normally compared to non-early-cleavage embryos (IVF: 69.1 % vs. 47.1 %, respectively; ICSI: 63.0 % vs. 45.6 %, respectively). The early-cleavage embryos also produced more good quality embryos than the non-early-cleavage embryos (IVF: 80.2 % vs. 56.4 %, respectively; ICSI: 73.4 % vs. 59.4 %). The implantation rate was significantly higher with early-cleavage embryos in both IVF (42.9 % vs. 19.7 %) and ICSI (48.1 % vs. 24 %). These results indicate that early-cleavage embryos have a higher rate of normal development and develop into better quality embryos on day 3, resulting in more and higher quality embryos to choose from for day-3 embryo transfer. Thus, early cleavage may be a useful criterion when selecting embryos for IVF or ICSI.

## Summary

Embryo selection is a difficult task on the embryo transfer day. Many parameters can be used to make this decision, including useful tools such as pronuclear morphology, Early cleavage (EC), blastomere morphology, and blastocyst grading. The clinical outcome of EC embryos should be evaluated when using the elective single embryo transfer (eSET) and double embryo transfer (DET) methods. In our study, we proved that for IVF, EC embryos (without mixed embryos) develop more normal cleavage and good quality embryos on day 3, and the selection of these embryos during IVF results in better clinical outcomes. Therefore, for IVF, identifying EC embryos is a useful tool for embryo selection. For the ICSI group, a better implantation rate was found by using EC embryos; the differences in the pregnancy and live birth rates were not statistically significant. Therefore, for ICSI, EC embryos should be identified 2 h earlier than for IVF to make this predictive marker more accurate. The use of EC as a marker for improving rates of pregnancy and live birth should be further investigated in ICSI cycles.

## Introduction

The identification of viable embryos is important for assisted reproductive therapy. Many embryo scoring or grading systems have been developed for choosing the best and most viable embryos. Oocyte and pronuclei morphology, blastomere number and symmetry, fragmentation, and blastocyst morphology are the usual criteria for selecting the best embryos at each stage [1, 2, 18]. Furthermore, several studies have shown that early cleavage (EC), defined as being 25–27 h after in vitro fertilization (IVF)/intracytoplasmic sperm injection (ICSI), is a strong indicator of embryo viability [2, 4–8].

In a previous study [9], the relationship between EC and day-2 embryo scores was evaluated; it was found that embryos with even EC developed into good day-2 embryos more often than those at the pronuclei or syngamy stages (ICSI: 69 % vs. 34 % and 29 %, respectively; IVF: 53 % vs. 37 % and 29 %, respectively). Even and mononucleated EC embryos had higher day-3 scores [8]. The blastocyst formation rate of EC embryos was higher than that of non-EC embryos (66 % vs. 40 %; 32.2 % vs. 16.6 %) [10, 11].

Many studies have compared EC embryos to non-EC embryos without distinguishing between IVF or ICSI cycles [3–5, 7, 12, 14–16]. In theory, the oocyte is fertilized earlier by the sperm in ICSI compared with IVF treatment because the spermatozoon is directly injected into the oocyte. Therefore, the EC rate should be higher in the ICSI cycle than the IVF cycle (34.7 % vs. 21.5 %) [3]. Van Montfoort et al. [7] evaluated EC 2 h earlier in the ICSI cycle. In this study, the outcomes of EC embryos were evaluated in both the IVF and ICSI cycles.

Furthermore, the outcome of EC embryo implantation should be evaluated when the elective single embryo transfer (eSET) method is used. Transferring mixed embryos (EC and non-EC) would affect the predictive value of EC. Some studies have evaluated the predictive value of EC after using mixed EC and non-EC embryos in IVF and ICSI cycles [3, 4, 17]. Other studies have evaluated the predictive value of EC using eSET but did not differentiate between IVF and ICSI cycles [5, 7, 14, 15]. These studies found that EC embryos had higher implantation and pregnancy rates. However, no studies have evaluated EC embryos using eSET in only IVF treatment cycles. Therefore, we selected treatment cycles that did not transfer mixed EC and non-EC embryos together in order to evaluate the outcomes of EC embryos.

The aim of this study was to assess the relationship between EC and day-3 embryonic blastomere number and quality in IVF and ICSI cycles. The rates of implantation, clinical pregnancy, and live birth were also recorded to evaluate the outcomes of EC embryo transfer in both cycles.

## Materials and methods

### Study subjects

This study was a retrospective study of IVF/ICSI outcomes based on the medical records of 185 patients (112 IVF cycles and 82 ICSI cycles) in the IVF laboratory of Mackay Memorial Hospital from December 2007 to November 2008. To minimize age-related effects, patients over 40 years of age were excluded from the study.

### Ovarian stimulation protocols

Two stimulation protocols were used: the gonadotropin-releasing hormone (GnRH) agonist long protocol (GnRH agonist group) and the GnRH antagonist protocol (GnRH antagonist group). In the GnRH agonist group, 0.5 mg (0.1 cc) of leuprolide acetate (Lupron, Takeda, Germany) was administered daily, commencing on day 21 of the previous menstrual cycle. Once serum levels of estradiol (E2) < 40 pg/ml were achieved, recombinant follicle-stimulating hormone (FSH; Gonal-F; Serono, Switzerland) and human menopausal gonadotropin (hMG; Menopur; Ferring, Germany) were administered from the third day of menstruation until the day of human chorionic gonadotropin (hCG) administration. The doses were adjusted according to each patient’s ovarian response. In the GnRH antagonist group, recombinant FSH and hMG were administered daily from the third day of the menstrual cycle. The doses were also adjusted according to each patient’s individual ovarian response. Once the dominant follicle reached 14 mm in mean diameter, 0.25 mg cetrorelix acetate (Cetrotide; Serono, Germany) was administered subcutaneously daily until the day of hCG administration.

In both groups, final maturation was induced using ether recombinant hCG (Ovidrel; Serono, Italy) or urinary hCG (Profasi; Serono, Switzerland) when at least 2 leading follicles reached 18 mm in diameter; oocyte retrieval was performed 34–36 h later. On day 3, the embryos were graded morphologically, and embryo transfer was performed 72 h after oocyte retrieval. In each stimulation protocol, patients in whom only EC embryos were transferred were designated the “early-cleavage” subgroup. Patients in whom no EC embryos were transferred were designated as the “non-early-cleavage” subgroup.

The luteal phase was supported by vaginal supplementation of Crinone (8 % progesterone, 1.125 g applicator; Serono, UK) or intramural injection of 50 mg progesterone once a day. To assess treatment outcome, serum hCG was measured 14 days after oocyte retrieval. Clinical pregnancy was defined as the observation of a gestational sac in the uterus by transvaginal ultrasonography. The live birth rate was defined as the live birth of one or more neonates.

### Insemination and ICSI

Male factor infertility patients with a sperm concentration of <10 × 10^6^/mL, motility <25 %, or normal morphologic sperm (Kruger’s strict criteria) <4 % received ICSI as treatment, whereas those without male factor infertility received conventional IVF treatment.

Either IVF or ICSI was performed 3–5 h after oocyte aspiration. For the IVF procedure, each oocyte was inseminated with 20 × 10^3^ motile spermatozoa in a single drop of 20 μl medium (Quinn’s Advantage Fertilization medium; SAGE IVF, Trumbull, CT, USA). For the ICSI procedure, 1–2 μl washed spermatozoa was placed in 7 % polyvinylpyrrolidone (PVP; SAGE IVF) and the sperm was injected using standard techniques. Each embryo was cultured in a single drop of 20 μl medium (Quinn’s Advantage Cleavage medium; SAGE IVF) supplemented with 10 % synthetic serum substitute (Quinn’s Advantage Serum Protein Substitute; SAGE IVF) that was covered with mineral oil (SAGE IVF) in an atmosphere of 5 % O_2_, 5 % CO_2_, and 90 % N_2_ at 37 °C.

### Assessment of fertilization, early cleavage, and embryo quality

Normal fertilization was confirmed by the presence of 2 pronuclei and 2 polar bodies 16–20 h (day 1) after IVF or ICSI. On the same day, EC examination was performed 25–27 h after IVF or ICSI. Embryos displaying 2 cells at inspection were considered EC embryos, whereas those that had not yet cleaved to the 2-cell stage were designated non-EC embryos. Embryos were further examined for their quality at 66–68 h (day 3) after IVF or ICSI. Day-3 embryos were classified according to blastomere number as follows: rapid cleavage (≥9 cells), normal cleavage (7–8 cells), or slow cleavage (≤6 cells). On the basis of quality, day-3 embryos were grouped into good embryos (<20 % fragmentation and an even blastomere) or poor embryos (>20 % fragmentation or an uneven blastomere).

## Statistical analysis

Statistical analysis was performed using SPSS software version 12.0 for Windows. The differences in means between 2 variables were calculated using the Mann–Whitney *U* test and unpaired *t* test. The differences in the rates of implantation, clinical pregnancy, abortion, live birth, and EC were calculated using the chi-square test. A *P* value of <0.05 was considered statistically significant.

## Results

A total of 194 treatment cycles were analyzed, 112 using IVF and 82 using ICSI. The cycle characteristics for IVF or ICSI are shown in Table 1. The age differences of patients who received IVF or ICSI were not statistically significant (34.0 ± 3.34 vs. 33.4 ± 3.60, respectively). There were 699 2-pronuclear (2PN) zygotes in the IVF cycles and 522 2PN zygotes in the ICSI cycles. The EC rate was higher in ICSI than IVF (36.8 % vs. 31.0 %, respectively; *P* = 0.041).

**Table 1:** Comparisons of the early cleavage rate and the non-early cleavage rate for IVF and ICSI cycles. NS: no statistically significantly

	IVF	ICSI	*P* value
No. of cycles	112	82	
Age (mean ± SD)	34.0 ± 3.34	33.4 ± 3.6	NS
Total 2PN	699	522	
Early cleavage (%)	217 (31.0)	192 (36.8)	0.041
Non-early cleavage (%)	482 (69.0)	330 (63.2)	0.041

The relationship between EC and day-3 embryonic development in IVF and ICSI cycles was assessed (Tables 2 and 3, respectively). For IVF, there were 217 embryos in the EC group and 482 embryos in the non-EC group. The normal cleavage rate was 69.1 % for EC embryos and 47.1 % for non-EC embryos (*P* < 0.05). The incidence of good embryos was 80.2 % in the EC group and 56.4 % in the non-EC group (*P* < 0.05). For ICSI, the EC group had 192 embryos and the non-EC group had 330 embryos. The normal cleavage rate was 63.0 % for the EC group and 45.6 % in the non-EC group (*P* < 0.05). The incidence of good embryos was 73.4 % for the EC group and 59.4 % for the non-EC group (*P* < 0.05).

**Table 2:** Embryonic development and quality of embryos in the IVF group

Embryonic development	EC	Non-EC	*P* value
No. of embryos:	217	482	
≥9 cells, rapid cleavage (%)	35 (16.1)	41 (8.5)	<0.05
7–8 cells, normal cleavage (%)	150 (69.1)	227 (47.1)	<0.05
≤6 cells, slow cleavage (%)	32 (14.7)	214 (44.4)	<0.05
Quality of embryos:			
Good embryos (%)	174 (80.2)	272 (56.4)	<0.05
Poor embryos (%)	43 (19.8)	210 (43.6)	<0.05

**Table 3:** The embryonic development and the quality of embryos in ICSI group

Embryonic development	EC	Non-EC	*P* value
No. of embryos:	192	330	
≥9 cells, rapid cleavage (%)	31 (16.1)	28 (8.5)	<0.05
7–8cells, normal cleavage (%)	121 (63.0)	149 (45.6)	<0.05
≤6 cells, slow cleavage (%)	40 (20.8)	153 (46.4)	<0.05
Quality of embryos:			
Good embryos (%)	141 (73.4)	196 (59.4)	<0.05
Poor embryos (%)	51 (26.6)	134 (40.6)	<0.05

In Tables 4 and 5, “EC group” implies that only EC embryos were transferred to the uterus, and “non-EC group” implies that only non-EC embryos were transferred to the uterus. Using IVF, 16 cycles were performed for the EC group and 48 cycles for the non-EC group. For the IVF cycles, there was no statistical significance with regard to the duration of stimulation, number of embryos transferred, or abortion rate. The peak E2 level (*P* < 0.05), number of oocytes retrieved (*P* < 0.05), number of mature oocytes (*P* < 0.05), number of 2PN zygotes (*P* < 0.05), clinical pregnancy rate (*P* < 0.05), and implantation rate (*P* < 0.05) were significantly higher in the EC group than in the non-EC group. The live birth rate was significantly higher in the EC group than in the non-EC group (62.5 % vs. 25.0 %, *P* < 0.05). For the ICSI cycles, there was no statistical significance with regard to age, duration of stimulation, FSH dosage, peak E2 level, number of oocytes retrieved, number of mature oocytes, number of 2PN zygotes, number of embryos transferred, or abortion rate. The implantation rate was statistically higher in the EC group than in the non-EC group (48.1 % vs. 24.0 %, *P* = *P* < 0.05). The live birth rate was higher in the EC group than in the non-EC group (54.5 % vs. 37.1 %) but was not statistically significant.

**Table 4:** Comparison of transferred embryos with only early-cleavage and non-early-cleavage embryos for the IVF group

	EC	Non-EC	*P* value
No. of cycles	16	48	
Age range (years; mean ± SD)	32.1 ± 3.2	34.1 ± 3.4	<0.05
Duration of stimulation (days)	11.06 ± 1.3	11.52 ± 2.4	0.71
FSH dosage	1890.00 ± 1160.2	2649.27 ± 1221.0	<0.05
Peak E2 level	3011.56 ± 1324.4	1781.98 ± 1167.2	<0.05
No. of oocyte retrieved	13.43 ± 5.3	7.56 ± 4.7	<0.05
No. of mature oocytes	12.44 ± 5.5	6.50 ± 4.4	<0.05
No. of 2PN (total)	9.50 ± 5.6	4.45 ± 3.3	<0.05
No. of embryos transferred	2.62 ± 0.7	2.65 ± 1.0	0.89
Clinical pregnancies (%)	12/16 (75)	18/48 (37.5)	<0.05
Implantations (%)	18/42 (42.9)	25/127 (19.7)	<0.05
Abortions (%)	3/12 (25)	2/18 (11.1)	0.36
Live births (%)	10/16 (62.5)	12/48 (25.0)	<0.05

**Table 5:** Comparison of transferred embryos with only early cleavage and non-early cleavage in the ICSI group

	EC	Non-EC	*P* value
No. of cycles	22	35	
Age range (years; mean ± SD)	32.77 ± 3.1	33.34 ± 4.1	0.49
Duration of stimulation (days)	11.68 ± 2.1	11.49 ± 1.8	0.81
FSH dosage	1969.09 ± 1382.4	2521.57 ± 1261.8	<0.05
Peak E2 level	2185.18 ± 1591.5	2306.94 ± 1619.8	0.75
No. of oocyte retrieved	10.86 ± 5.25	10.14 ± 5.93	0.55
No. of mature oocytes	9.27 ± 4.8	8.26 ± 5.1	0.47
No. of 2PN (total)	6.73 ± 4.6	6.06 ± 4.29	0.61
No. of embryos transferred	2.45 ± 0.7	2.74 ± 1.0	0.18
Clinical pregnancies (%)	15/22 (68.2)	18/35 (51.4)	0.27
Implantations (%)	26/54 (48.1)	23/96 (24.0)	<0.05
Abortions (%)	3/15 (20.0)	5/18 (27.8)	0.70
Live births (%)	12/22 (54.5)	13/35 (37.1)	0.27

## Discussion

Many factors can influence the outcomes of IVF treatment or ICSI, but embryo morphology is the most common and useful tool in selecting the best embryos for transfer. Because eSET and double embryo transfer (DET) procedures have been recently recommended, choosing good quality embryos to enhance the rates of implantation, pregnancy, and live birth is very important. The use of EC identification to select embryos in humans was first reported by Edwards et al. [13]. Several studies have confirmed that EC is a strong indicator for viable embryo selection in humans [3–11]. In particular, using the eSET method with EC embryos results in higher implantation and pregnancy rates than those obtained using the eSET method with non-EC embryos [5, 7, 15]. The influence of EC on the live birth rate has been controversial. Emiliani et al. [14] claimed that assessing EC did not improve the delivery rate of single embryo transfer, although Lundin et al. [3] showed that EC was an independent predictor of birth in ICSI cycles.

Day-1 embryo development may be assessed by observing pronuclear morphology or EC, and day-3 morphology may be assessed by evaluating the number and morphology of blastomeres, including the percentage of fragmentation and even blastomeres. Terriou et al. [9] claimed that even EC embryos are strongly associated with good embryo morphology (53 % in IVF, 69 % in ICSI). EC embryos also show higher rates of blastocyst formation (66 % in both IVF and ICSI) [10]. We evaluated the correlation between EC embryos and day-3 embryo development. Normal cleavage rates were 69.1 % for IVF and 63.0 % for ICSI. Good embryo rates were 80.2 % for IVF and 73.4 % for ICSI. These results were statistically significantly higher than those obtained using non-EC embryos. Therefore, for both IVF and ICSI, more EC embryos developed into embryos with a normal cleavage rate and good morphology on day 3. These identified embryos should be selected for transfer to enhance the pregnancy rate.

The outcomes of using EC embryos were also evaluated by assessing the rates of clinical pregnancy, implantation, abortion, and live birth. The EC group had statistically higher implantation rates than the non-EC group in both the IVF and ICSI cycles. There was no difference in the abortion rate between the groups for the 2 cycles. Interestingly, the clinical pregnancy and live birth rates were statistically significantly higher for the EC group than the non-EC group for the IVF cycles but not statistically higher for the ICSI cycles.

The data showing the relationship among EC embryos, day-3 embryonic development, and clinical outcomes were divided into the IVF and ICSI groups (Tables 2 and 3, respectively). Because the spermatozoon is injected into the oocyte in the ICSI procedure, the zona pellucida, cumulus, and corona cell barrier are overcome. This process of fertilization provides the ICSI embryos a temporal advantage of approximately 2–4 h compared with the IVF procedure. The EC rate is higher in ICSI than in IVF (36.8 % vs. 31.0 %, respectively). Thus, the examination of EC should be performed approximately 23–25 h after ICSI to improve the accuracy rate of pregnancy prediction in the ICSI group [7]. We separated the assisted fertilization cases into 2 groups, IVF and ICSI, to investigate the effect of EC on both groups. The predictive value of using EC embryos as an indicator of pregnancy outcome is relatively weak, as mentioned by Van Montfoort et al. [7]. They reported that many other oocyte and embryonic factors may influence the implantation capacity of the embryo. In addition, the pregnancy outcome is not only dependent on embryo quality but also on endometrial receptivity.

Embryo selection is a difficult task on the embryo transfer day. Many parameters can be used to make this decision, including useful tools such as pronuclear morphology, EC, blastomere morphology, and blastocyst grading. The clinical outcome of EC embryos should be evaluated when using the eSET and DET methods. For both IVF and ICSI cycles, the effect of EC on embryo transfer using mixed embryos (including EC and non-EC embryos) could not be accurately evaluated. In our study, we proved that for IVF, EC embryos (without mixed embryos) develop more normal cleavage and good quality embryos on day 3, and the selection of these embryos during IVF results in better clinical outcomes. Therefore, for IVF, identifying EC embryos is a useful tool for embryo selection. For the ICSI group, a better implantation rate was the only clinical outcome using EC embryos; the differences in the pregnancy and live birth rates were not statistically significant. Therefore, for ICSI, EC embryos should be identified 2 h earlier than for IVF to make this predictive marker more accurate. The use of EC as a marker for improving rates of pregnancy and live birth should be further investigated in ICSI cycles.
